# Expression of the normal epithelial cell-specific 1 (*NES1*; KLK10) candidate tumour suppressor gene in normal and malignant testicular tissue

**DOI:** 10.1054/bjoc.2001.1870

**Published:** 2001-07

**Authors:** L-Y Luo, E Rajpert-De Meyts, K Jung, E P Diamandis

**Affiliations:** Department of Pathology and Laboratory Medicine, Mount Sinai Hospital, 600 University Avenue, Toronto, Ontario, M5G 1X5, Canada; Department of Laboratory Medicine and Pathobiology, University of Toronto, 110 College Street, Toronto, Ontario, M5G IL5, Canada; Department of Growth and Reproduction, Juliane Marie Centre, National University Hospital (Rigshospitalet), Copenhagen, Denmark; Department of Urology, University Hospital Charite, Humboldt University, Berlin, Germany

**Keywords:** normal epithelial cell-specific 1 (*NES1*) gene, KLK10, kallikrein, testicular cancer, down-regulation, tumour suppressor genes

## Abstract

The normal epithelial cell-specific 1 (*NES1*) gene (official name kallikrein gene 10; KLK10) is a new member of the expanding human kallikrein gene family and encodes for a secreted serine protease. Experimental evidence suggests that NES1 controls normal cell growth and may function as a tumour suppressor. NES1 is down-regulated during breast cancer progression. The *NES1* gene is highly expressed in testicular as well as in other tissues. In this study, we investigated the expression level of the *NES1* gene in cancerous and normal testicular tissues with reverse transcriptase-polymerase chain reaction (RT-PCR) and immunohistochemistry. In all 14 primary testicular germ-cell tumours examined, the *NES1* gene expression was markedly reduced compared to adjacent (paired) normal tissues. We further examined 6 randomly selected primary germ-cell tumours and 8 normal tissues (obtained from different individuals). We confirmed the differential expression of the *NES1* gene in germ-cell tumours (GCT) and pre-malignant carcinoma in situ (CIS). Our findings suggest that NES1 may act as a tumour suppressor and may play a role in the pathogenesis and progression of this malignancy. © 2001 Cancer Research Campaign http://www.bjcancer.com


*British Journal of Cancer* (2001) **85**(2), 220–224
© 2001 Cancer Research Campaign
doi: 10.1054/ bjoc.2001.1870, available online at http://www.idealibrary.com on IDEAL®

### Expression of the normal epithelial cell-specific 1 (*NES1*; KLK10) candidate tumour suppressor gene in normal and malignant testicular tissue

**L-Y Luo[1,2], E Rajpert-De Meyts[3], K Jung[4] and EP Diamandis[1,2]**

[1]Department of Pathology and Laboratory Medicine, Mount Sinai Hospital, 600 University Avenue, Toronto, Ontario M5G 1X5, Canada; [2]Department of Laboratory Medicine and Pathobiology, University of Toronto, 110 College Street, Toronto, Ontario M5G IL5, Canada; [3]Department of Growth and Reproduction, Juliane Marie Centre, National University Hospital (Rigshospitalet), Copenhagen, Denmark; [4]Department of Urology, University Hospital Charite, Humboldt University, Berlin, Germany

**Summary** The normal epithelial cell-specific 1 (*NES1*) gene (official name kallikrein gene 10; KLK10) is a new member of the expanding human kallikrein gene family and encodes for a secreted serine protease. Experimental evidence suggests that NES1 controls normal cell growth and may function as a tumour suppressor. NES1 is down-regulated during breast cancer progression. The *NES1* gene is highly expressed in testicular as well as in other tissues. In this study, we investigated the expression level of the *NES1* gene in cancerous and normal testicular tissues with reverse transcriptase-polymerase chain reaction (RT-PCR) and immunohistochemistry. In all 14 primary testicular germ-cell tumours examined, the *NES1* gene expression was markedly reduced compared to adjacent (paired) normal tissues. We further examined 6 randomly selected primary germ-cell tumours and 8 normal tissues (obtained from different individuals). We confirmed the differential expression of the *NES1* gene in germ-cell tumours (GCT) and pre-malignant carcinoma in situ (CIS). Our findings suggest that NES1 may act as a tumour suppressor and may play a role in the pathogenesis and progression of this malignancy. © 2001 Cancer Research Campaign http://www.bjcancer.com

**Keywords**: normal epithelial cell-specific 1 (*NES1*) gene; KLK10; kallikrein; testicular cancer; down-regulation; tumour suppressor genes

The normal epithelial cell-specific 1 (*NES1*) gene (also known as KLK10, based on the official nomenclature) resides on chromosome 19q13.3–4, spans about 5.5 kb of genomic DNA sequence and contains 6 exons and 5 introns. *NES1* encodes for a secreted serine protease, whose amino acid sequence has 35–40% identity and 50–55% similarity with other members of the human kallikrein family (including human kallikrein 1, human kallikrein 2, and prostate specific antigen) (Liu et al, 1996; Luo et al, 1998). Thus, the *NES1* gene is considered to be a new member of this family (Diamandis et al, 2000). The physiological function of NES1 is still not clear. The *NES1* gene was identified by virtue of its down-regulation in breast cancer cell lines, and it is considered to be involved in the regulation of normal cell growth. Further experimental evidence suggests that NES1 may function as a tumour suppressor gene. When the *NES1* gene was transfected into the tumorigenic breast cancer cell line MDA-MB-231, its anchorage-independent growth was reduced and when this cell line was inoculated into nude mice, tumour formation was significantly decreased (Goyal et al, 1998).

*NES1* is highly expressed in testicular tissue (Liu et al, 1996). Based on the observations in breast cancer, we hypothesized that the expression of this gene might also be altered in testicular tumours. Testicular cancer is the most common malignancy of young males. In North America, its annual incidence rate is 3–4/100 000 (Coleman et al, 1993). About 90% of the testicular tumours originate from germ-cell tissues (germ-cell tumours, GCTs) and only 10% are derived from interstitial tissues (non-germ-cell tumours), such as the rare Leydig cell tumour. According to the World Health Organization, testicular GCTs are classified into seminomas and nonseminomas. Nonseminomas are further divided into various subgroups based on their histologic types, including embryonal carcinoma, teratoma, choriocarcinoma, and other tumours made up of more than one histologic type. GCTs are malignant tumours, except for mature teratoma which often has a more benign clinical course (Einhorn, 1994). GCTs originate from a common pre-invasive lesion, carcinoma in situ, CIS (Skakkebaek, 1972; Skakkebaek et al, 1987). The events involved in initiation and progression from normal germ cells to CIS, then to malignant GCTs are still unknown. It is believed that aberrant gonadal development may be responsible for the origin of CIS (Skakkebaek et al, 1987; Rajpert-De Meyts et al, 1998), whereas both oncogenes and tumour suppressor genes contribute to the invasive transformation to overt tumours (Dean and Moul, 1998; Looijenga and Oosterhuis, 1999).

In this study, we investigated the expression of *NES1* in normal and malignant testicular tissues at the RNA and protein level, and found differential expression of this gene during progression of germ cell neoplasia.

*Received 20 September 2000*
*Revised 2 January 2001*
*Accepted 11 January 2001*

*Correspondence to:* EP Diamandis

### MATERIALS AND METHODS

### Testicular specimens

14 matched samples of tumours and adjacent morphologically normal testicular tissues were obtained from patients who had undergone radical orchiectomy at the University Hospital Charite, Berlin, Germany. Patient age ranged from 23–60 years with a median of 36. All patients had a histologically confirmed diagnosis of primary testicular germ cell cancer and received no treatment before surgery. The samples included 6 seminomas and 8 non-seminomas (teratomas, embryonal carcinomas and choriocarcinomas). 6 randomly selected seminomas (without matched normal tissues) were also included. 8 normal testicular tissue specimens were obtained from patients who had orchiectomy for prostate cancer. Subsequently, 6 additional testicular tumour specimens that included CIS and Leydig tumour, and 2 human embryonal carcinoma cell lines, NTERA-2 and 2102Ep were obtained from the National University Hospital, Copenhagen, Denmark.

### Total RNA extraction

Tissue specimens were minced with a scalpel, on ice, and immediately transferred into a 2 ml polypropylene tube. They were then homogenized and total RNA was extracted using RNeasy mini spin columns (QIAGEN Inc., Valencia, CA) following the manufacturer's instructions. Total RNA concentration and its purity were determined spectrophotometrically.

### Reverse transcriptase-polymerase chain reaction (RT-PCR)

2 μg of total RNA were converted to cDNA with the Superscript™ Preamplification Kit (Gibco BRL, Gaithersburg, MD), following the manufacturer's recommendations. The final volume was 20 μl. 2 PCR primers were designed to amplify the *NES1* cDNA. Their sequences are as follows: forward, 5′-GATCACCTGCT-GCTTCTTC-3′; reverse, 5′-CACTCTGGCAAGGGTCCTG-3′. The expected size of the PCR product is 383 bp. The actin primers are: forward, 5′-ACAATGAGCTGCGTGTGGCT-3′; reverse, TCTCCTTAATGTCACGCACGA-3′. The amplified PCR product is 372 bp. PCR was carried out in a 20 μl reaction mixture, containing 1 μl of cDNA, 10 mM Tris-HCl (pH 8.3), 50 mM KCl, 1.5 mM $MgCl_2$, 200 μM dNTPs (deoxynucleoside triphosphates), 100 ng primers and 2.5 units of HotstarTaq™ DNA polymerase (QIAGEN) on a Perkin-Elmer 9600 thermal cycler. In all PCR reactions, a blank control (water was used as the template) was always included. The same PCR conditions were used for both *NES1* gene and actin gene amplification. They were 94°C for 15 minutes to activate the polymerase, followed by 30 cycles of denaturing at 94°C for 30 seconds, annealing at 62°C for 30 seconds, and extension at 72°C for 30 seconds. The final extension was at 72°C for 5 minutes. Equal amounts of PCR products were then separated on a 2.5% agarose gel, stained with ethidium bromide, and visualized under UV light.

### Immunohistochemical localization of NES1 protein

A rabbit polyclonal antibody was raised against the *NES1* gene full-size recombinant protein produced in yeast cells. Immunohistochemical staining for NES1 was performed according to a standard indirect immunoperoxidase method. Briefly, frozen or fixed and paraffin-embedded tissue sections were thawed or fixed and dewaxed. The procedure was performed at room temperature (RT) unless otherwise specified. To block an unspecific antibody binding, 10% non-immune goat serum was applied to sections for 20 min. The endogenous activity of peroxidase was not blocked by an incubation with a hydrogen peroxide solution (which is normally a part of the protocol) because the specific staining was somewhat weaker after this pretreatment. However, it is known from numerous previous experiments that erythrocytes are the only cell type in the testis that exhibits endogenous peroxidase activity. Subsequently, the sections were incubated with the primary anti-NES1 antibody overnight at +4°C. For each specimen, a negative control was incubated with the Tris buffer instead of the primary antibody. After triple wash in Tris buffer, a biotinylated goat-anti-rabbit link antibody was applied, followed by a streptavidin-peroxidase conjugate (both from Zymed). The red colour at the site of reaction was visualized by adding a solution of acetyl carbazole (ACE) and hydrogen peroxide (Zymed). Finally, the sections were lightly counterstained with haematoxylin and mounted with cover slips using an aqueous, glycerol-based mounting medium (DAKO).

### RESULTS

In order to investigate the potential role of the *NES1* gene in testicular tumorigenesis, we examined *NES1* gene expression in the normal human testis, in a panel of testicular tumours and in 2 germ-cell-tumour derived cell lines. We examined the expression at the RNA level by RT-PCR and at the protein level by immunohistochemistry. To ensure that equal amounts of cDNA were used for RT-PCR in different samples, the actin gene was also amplified as a control. We first compared *NES1* gene expression in 14 paired tumour/normal testicular tissues. As shown in Figure 1, for all 14 pairs, the expression of *NES1* was abundant in normal testicular tissues, but was markedly lower or undetectable in the tumour samples. The actin gene (control) has similar expression level in tumours and adjacent tissues, indicating that the reduced *NES1* expression in cancerous tissues is not due to the amount of RNA used. We further examined *NES1* expression in 6 randomly

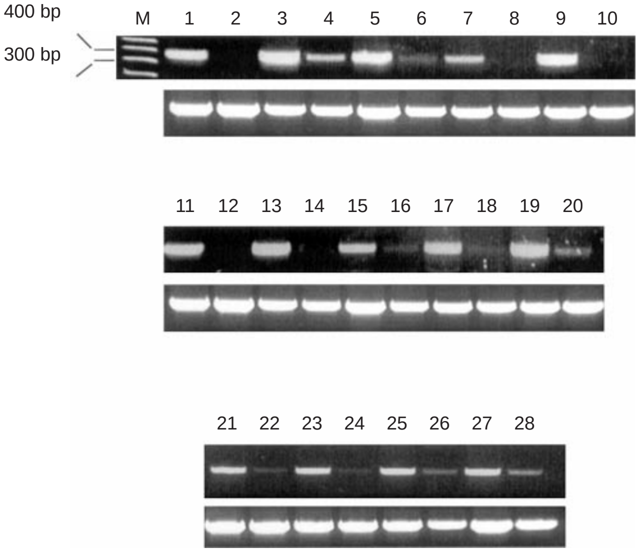

**Figure 1** *NES1* gene expression in paired cancerous/normal tissues as assessed by RT-PCR. The normal tissues were obtained from the adjacent non-cancerous parts in the same testis. The upper and lower panels show the PCR products of the *NES1* gene and actin gene, respectively. Lane M: molecular weight marker. Odd lanes: normal tissues. Even lanes: cancerous tissues (lanes 2, 4, 6, 10, 12, 14, 22, 28: non-seminomas; lanes 8, 16, 18, 20, 24, 26: seminomas)

© 2001 Cancer Research Campaign selected specimens of seminoma and 8 normal testicular tissues from different individuals (unpaired cancerous/normal tissues). For all 8 normal testicular tissues, the expression of *NES1* was uniformly high. However, for all 6 testicular tumours, *NES1* gene expression was either low or undetectable (Figure 2). These results further confirmed that the expression of *NES1* gene was high in the normal testis but it was markedly decreased in testicular GCTs.

Subsequently, we examined the expression of *NES1* in testicular tumours with different histologic types. One specimen with a widespread CIS (80–90% of CIS tubules, as established by a histological examination of adjacent tissue sections), and a Leydig cell tumour demonstrated nearly as high level of *NES1* expression as normal parenchyma, whereas seminoma, nonseminoma, and embryonal carcinoma cell lines had very low expression (Figure 3). This indicated that the *NES1* gene is down-regulated or not present in overt GCTs, but its expression is high in a non-germ cell testicular tumour (the Leydig cell tumour), and the tissue with CIS, which retains the architecture of testicular parenchyma. In the latter case, somatic cells are present, but the majority of normal germ cells are replaced by the pre-invasive CIS cells.

The interpretation of these data depended upon the cellular localization of NES1 protein. To that end, we have raised a polyclonal rabbit-anti-human NES1 antibody and performed immunohistochemical staining (Figure 4). In paraffin sections, the results of immunostaining differed somewhat depending upon the fixative used, however, in all fixatives, the normal testis showed a clear staining of germ cells, mainly spermatogonia. A diffuse staining was also observed in Sertoli cells, especially in areas adjacent to spermatogonia and the basement membrane of seminiferous tubules. A weak reaction was seen in some Leydig cells, mainly in sections fixed in Bouin's fluid. To eliminate the influence of fixatives, the immunostaining was also performed in frozen specimens, and the localization of the NES1 protein to spermatogonia (strong) and adjacent Sertoli cells (weaker) was confirmed (Figure 4). The sections with CIS which were obtained from the same orchiectomy specimen used earlier for the RT-PCR experiments, demonstrated a clear staining of CIS cells. Preliminary results

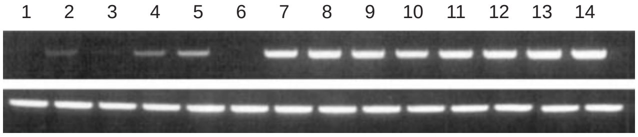

**Figure 2** *NES1* gene expression in unpaired cancerous and normal testicular tissues as assessed by RT-PCR. The upper and lower panels show the PCR products of the *NES1* gene and actin gene, respectively. Lane 1–6, cancerous testicular tissues (all seminomas). Lane 7–14, normal testicular tissues

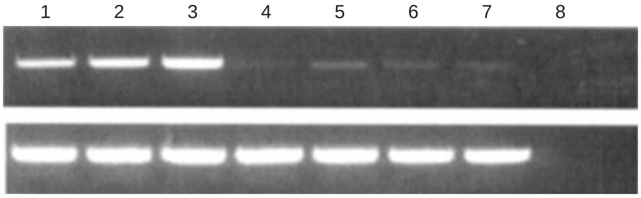

**Figure 3** The expression of *NES1* gene in normal testicular tissue and different histologic types of testicular tumour specimens. The upper and lower panels show the PCR products of the *NES1* gene and actin gene, respectively. Lane 1, carcinoma in situ; 2, normal parenchyma; 3, Leydig cell tumou; 4, seminoma; 5, non-seminoma; 6, embryonal carcinoma-derived NTERA-2 cell line (pluripotent); 7, embryonal carcinoma-derived 2102Ep cell line (nullipotent). 8, Blank control for PCR

from staining of a few overt tumours indicated that there may be a differential expression of the NES1 protein in germ cell tumours; seminomas appeared to be negative, whereas some highly differentiated elements present within mature teratomas were NES1-positive. That would explain the presence of detectable *NES1* expression in some tumours in the RT-PCR experiments (e.g. line 4 and 28 in Figure 1).

### DISCUSSION

In this study, we report for the first time that the *NES1* gene is differentially expressed in testicular germ-cell tumours, in comparison to normal testicular tissues. The mechanism underlying this finding is not clear, but this data are in accord with previous reports, which suggest that NES1 may function as a tumour suppressor gene (Liu et al, 1996; Goyal et al, 1998). 2 general mechanisms have been proposed for the down-regulation of tumour suppressor genes in cancer (Lee et al, 1991). First, deteriorate mutations in tumour suppressor genes could alter their expression. In testicular GCTs mutations have been reported exceedingly rarely. One example may be the tumour suppressor p16 (a cell cycle regulatory protein), which is found to be down-regulated in a proportion of testicular tumours. The *p16* gene exhibits high mutations frequency in tumour samples but not in matching normal controls (Heidenreich et al, 1998). Second, down-regulation of tumour suppressor genes may be due to suppression of their regulatory factors (transcriptional modulation). This mechanism is thought to contribute to the down-regulation of retinoblastoma (RB) gene in testicular cancer (Strohmeyer et al, 1991). A similar mechanism might also apply to the *NES1* gene for the following reasons: (a) The *NES1* gene is down-regulated in breast cancer cell lines, but no deletions or rearrangements of the *NES1* gene have been detected (Liu et al, 1996), (b) Chromosome 19q 13.3–4, the region where the *NES1* gene resides, is not prone to deletions or rearrangements in testicular cancer (Dean and Moul, 1998). However, one has to keep in mind that the testis and testicular GCTs differ from other tissues with regard to regulation of proliferation, mainly because of the unique biology of germ cells, which are capable of undergoing 2 types of cell division: mitotic and meiotic. Moreover, CIS cells and GCTs retain to a large extent embryonic-like pluripotency and may differentiate to a range of diverse somatic and even extra-embryonic tissues (Andrews, 1998). Thus, the results of investigations of gene expression in these tumours have to consider the delicate balance between cellular proliferation and differentiation.

In general, the expression of *NES1* in testicular GCTs was low or undetectable in overt tumours, both seminomas and non-seminomas, but a couple of tumour samples showed some expression at the RNA level. In the case of seminomas it may be explained by a possible presence of tubules with CIS (or even single non-malignant tubules) within the tumour tissue, which is frequently seen. The relatively highest NES1 expression was though seen in 2 non-seminomas. This was explained by the immunohistochemical studies at the protein level which clearly showed that in analogy to normal somatic epithelial tissues, some highly differentiated epithelial elements of teratomas and teratocarcinomas displayed a high level of NES1 expression. In contrast to invasive seminomas, the pre-invasive precursor of GCTs, carcinoma in situ (CIS), retained a high expression of NES1, similarly to normal spermatogonia. CIS cells have a similar phenotype to overt seminomas, and very few differences between these 2 cell types have been reported

 © 2001 Cancer Research Campaign

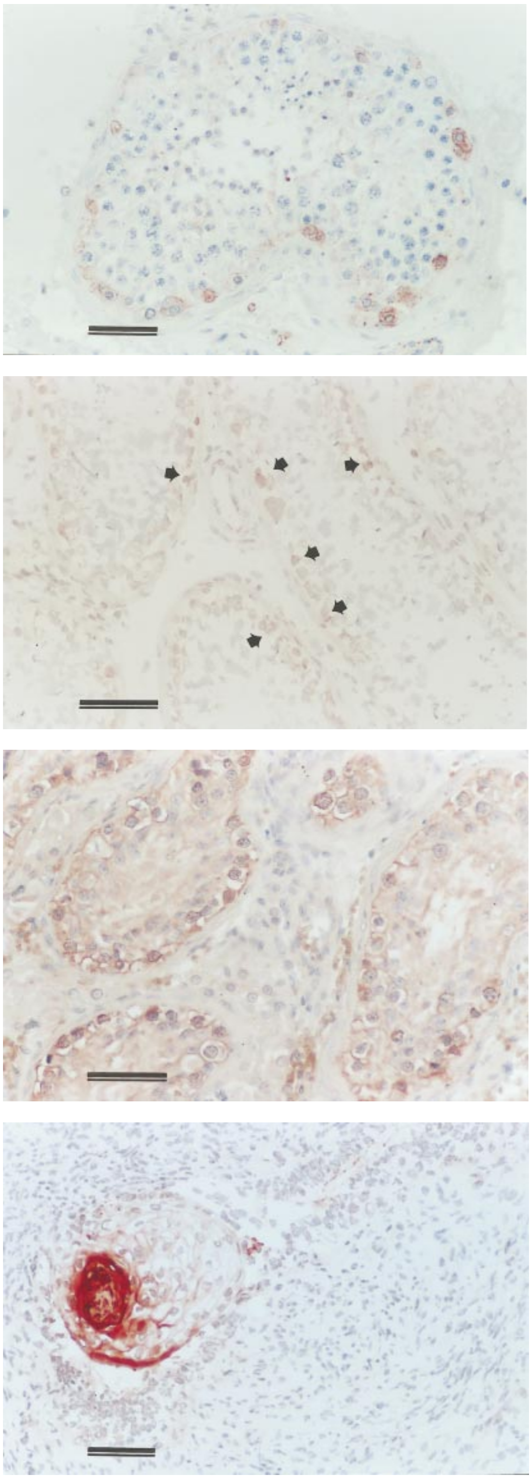

A B C D

**Figure 4** Cellular localization of the *NES1* gene protein product in normal and malignant testicular tissues. Scale bar = 50 µm. (**A**) A tubule from the normal testis. Note a strong reaction for NES1 in spermatogonia. (**B**) A frozen section of the normal testis demonstrating the strongest staining in spermatogonia (arrows). (**C**) A section with carcinoma in situ (CIS, a tissue sample adjacent to that examined by RT-PCR in Figure 3). There is a strong staining of CIS cells and diffuse staining of Sertoli cells. (**D**) An example of NES1 staining in teratoma. Note a high NES1 expression in a well-differentiated epithelial component, whereas the less differentiated parts of the tumour are negative so far. The most important among them is p18 (INK4c), an inhibitor of cyclin-dependent kinase, which is abundant in CIS but down-regulated in seminoma (Bartkova et al, 2000).

As far as the normal tesis is concerned, our study clearly demonstrated that the expression of the *NES1* gene was mainly localized in spermatogonia. There may be a low level of expression in Sertoli cells, or a diffusion of the secreted protein, but the weak staining observed in our preliminary immunohistochemical experiments was dependent on the method of tissue preservation, and may be a result of the cross-reaction with another protein. Further studies on a larger number of tissue samples will be necessary to draw definitive conclusions.

*NES1* is a new member of the kallikrein gene family. All kallikrein enzymes described so far are secreted proteins and some of them are valuable circulating cancer biomarkers (Diamandis et al, 2000). The most notable example is prostate specific antigen (PSA) (Stamey et al, 1987; Oesterling, 1991). PSA is a favourable marker for breast cancer prognosis (Yu et al, 1995). Also, the ratio between free PSA and human kallikrein 2 has potential for distinguishing prostate cancer from benign prostate hyperplasia (Magklara et al, 1999). Since NES1 is also a secreted protein, we speculate that it may be detectable in blood or semen samples, and may have value as a biomarker of testicular function and tumour progression.

In summary, in this study, we found that the *NES1* gene is expressed primarily by normal germ cells and is universally under-expressed in testicular germ-cell tumours. The high expression in some differentiated tumours suggests that transcriptional regulation rather than structural loss is the mechanism responsible for the low NES1 expression in germ-cell tumours. We hypothesize that the *NES1* gene expression may be linked to proliferation of germ cells and may play a role in progression of testicular cancer, consistent with a putative tumour suppressor function of this protein.

### ACKNOWLEDGEMENTS

The authors thank Professor Niels E Skakkebaek for useful comments. Part of the work was supported by The Danish Cancer Society.

### REFERENCES

Andrews PW (1998) Teratocarcinomas and human embryology: Pluripotent human EC lines. *APMIS* **106**: 158–168

Bartkova J, Thullberg M, Rajpert-De Meyts E, Skakkebaek NE and Bartek J (2000) Cell cycle regulators in testicular cancer: loss of p18$^{INK4C}$ marks progression from carcinoma *in situ* to invasive germ cell tumours. *Int J Cancer* **85**: 370–375

Coleman MP, Esteve J, Damiecki P, Arslan A and Renard H (1993) Trends in cancer incidence and mortality. *IARC Scientific Publications* **121**: 521–542

Dean RC and Moul JW (1998) New tumor markers of testis cancer. *Urologic Clinics of North America* **25**: 365–373

Diamandis EP, Yousef GM, Luo L, Magklara I and Obiezu CV (2000) The new human kallikrein gene family: implications in carcinogenesis. *Trends Endocrinol Metab* **11**: 54–60

Einhorn LH (1994) Salvage therapy for germ cell tumors. *Semin Oncol* **21**: 47–51

Goyal J, Smith KM, Cowan JM, Wazer DE, Lee SW and Band V (1998) The role for NES1 serine protease as a novel tumor suppressor. *Cancer Res* **58**: 4782–4786

Heidenreich A, Gaddipati JP, Moul JW and Srivastava S (1998) Molecular analysis of P16(Ink4)/CDKN2 and P15(INK4B)/MTS2 genes in primary human testicular germ cell tumours. *J Urol* **159**: 1725–1730

Lee SW, Tomasetto C and Sager R (1991) Positive selection of candidate tumor-suppressor genes by subtractive hybridization. *Proc Natl Acad Sci USA* **88**: 2825–2829

*© 2001 Cancer Research Campaign*

Liu XL, Wazer DE, Watanabe K and Band V (1996) Identification of a novel serine protease-like gene, the expression of which is down-regulated during breast cancer progression. *Cancer Res* **56**: 3371–3379

Looijenga LHJ and Oosterhuis JW (1999) Pathogenesis of testicular germ cell tumors. *Rev Reprod* **4**: 90–100

Luo L, Herbrick JA, Scherer SW, Beatty B, Squire J and Diamandis EP (1998) Structural characterization and mapping of the normal epithelial cell-specific 1 gene. *Biochem Biophys Res Commun* **247**: 580–586

Magklara A, Scorilas A, Catalona WJ and Dimandis EP (1999) The combination of human glandular kallikrein and free prostate-specific antigen (PSA) enhances discrimination between prostate cancer and benign prostatic hyperplasia in patients with moderately increased total PSA. *Clin Chem* **45**: 1960–1966

Oesterling JE (1991) Prostate specific antigen: a critical assessment of the most useful tumor marker for adenocarcinoma of the prostate. *J Urol* **145**: 907–923

Rajpert-De Meyts E, Jørgensen NE, Nielsen KB, Müller J and Skakkebæk NE (1998) Developmental arrest of germ cells in the pathogenesis of germ cell neoplasia. *APMIS* **106**: 198–206

Skakkebaek NE (1972) Possible carcinoma in situ of the testis. *Lancet* **ii**: 516–517

Skakkebaek NE, Berthelsen JG, Giwercman A and Muller J (1987) Carcinoma-in-situ of the testis: possible origin from gonocytes and precursor of all types of germ cell tumours except spermatocytoma. *Int J Androl* **10**: 19–28

Stamey TA, Yang N, Hay AR, McNeal JE, Freiha FS and Redwine E (1987) Prostate-specific antigen as a serum marker for adenocarcinoma of the prostate. *N Engl J Med* **317**: 909–916

Strohmeyer T, Reissmann P, Cordon-Cardo C, Hartmann M, Ackermann R and Slamon D (1991) Correlation between retinoblastoma gene expression and differentiation in human testicular tumors. *Proc Natl Acad Sci USA* **88**: 6662–6666

Yu H, Giai M, Diamandis EP, Katsaros D, Sutherland DJ, Levesque MA, Roagna R, Ponzone R and Sismondi P (1995) Prostate-specific antigen is a new favorable prognostic indicator for women with breast cancer. *Cancer Res* **55**: 2104–2110

*© 2001 Cancer Research Campaign*
